# A pain-inducing centipede toxin targets the heat activation machinery of nociceptor TRPV1

**DOI:** 10.1038/ncomms9297

**Published:** 2015-09-30

**Authors:** Shilong Yang, Fan Yang, Ningning Wei, Jing Hong, Bowen Li, Lei Luo, Mingqiang Rong, Vladimir Yarov-Yarovoy, Jie Zheng, KeWei Wang, Ren Lai

**Affiliations:** 1 Key Laboratory of Animal Models and Human Disease Mechanisms, Chinese Academy of Sciences, Kunming Institute of Zoology, Kunming 650223, Yunnan, China; 2 University of Chinese Academy of Sciences, Beijing 100009, China; 3 Department of Physiology and Membrane Biology, University of California, Davis, California 95616, USA; 4 Department of Neurobiology, Neuroscience Research Institute, Peking University Health Science Center, Beijing 100191, China; 5 College of Biological Science and Engineering, Fuzhou University, Fuzhou 350116, China; 6 Department of Molecular and Cellular Pharmacology, PKU-IDG/McGovern Institute for Brain Research, Peking University School of Pharmaceutical Sciences, Beijing 100191, China; 7 Department of Pharmacology, Qingdao University, Qingdao 266021, China

## Abstract

The capsaicin receptor TRPV1 ion channel is a polymodal nociceptor that responds to heat with exquisite sensitivity through an unknown mechanism. Here we report the identification of a novel toxin, RhTx, from the venom of the Chinese red-headed centipede that potently activates TRPV1 to produce excruciating pain. RhTx is a 27-amino-acid small peptide that forms a compact polarized molecule with very rapid binding kinetics and high affinity for TRPV1. We show that RhTx targets the channel’s heat activation machinery to cause powerful heat activation at body temperature. The RhTx–TRPV1 interaction is mediated by the toxin’s highly charged C terminus, which associates tightly to the charge-rich outer pore region of the channel where it can directly interact with the pore helix and turret. These findings demonstrate that RhTx binding to the outer pore can induce TRPV1 heat activation, therefore providing crucial new structural information on the heat activation machinery.

## Abstract (web summary)

The venom of the Chinese red-headed centipede causes excruciating pain. Here, Yang *et al*. identify a novel toxin protein from the centipede venom and find that it can activate the nociceptive TRPV1 ion channel by binding to the channel’s outer pore to potentiate the heat activation machinery.

## Graphical abstract

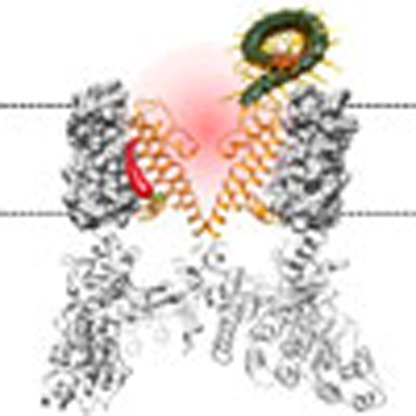

Venomous animals use toxins to paralyse prey for hunting or to inflict pain during self-defence^1^. A large fraction of the known nerve toxins from spider, snake, scorpion, sea anemone and cone snail (the 5S’s) achieve these purposes by targeting ion channels^2^,^3^. Binding of animal toxins either blocks ion permeation or interferes with activation gating, therefore disrupting the normal function of their targets. For example, snake α-bungarotoxin inhibits nicotinic acetylcholine receptor of neuromuscular junction, causing respiratory paralysis and death. Scorpion charybdotoxin and spider hanatoxin inhibit voltage-gated Kv channels, causing hyper-excitability of the nervous system. The action of most known animal toxins is inhibitory in nature^2^,^3^. Noticeably, spider toxins VaTx^4^ and DkTx^5^ are found to activate nociceptor TRPV1 ion channel, hence representing a unique defence mechanism.

Many of the 3,000 centipede species are highly venomous^6^,^7^; their bites are known to kill small animals such as rodent, snake and even human^8^,^9^. Centipede bites are characterized by extremely sharp pain that has an instant onset and lasts from half an hour up to 2–3 days^10^. Centipede venoms are generally not as lethal to human and other vertebrates as some snake or scorpion venoms; however, the distinctive algogenic property has clear defensive significance for these terrestrial, near-blind creatures^11^. How toxins in centipede venom interact with the victim body has just begun to be understood^6^,^7^.

Here we report the discovery of a novel peptide toxin from the Chinese red-headed centipede *Scolopendra subspinipes mutilans*, a bright-coloured aggressive predator up to 20 cm in length (Fig. 1a). The toxin, named here RhTx, was found to target TRPV1, which is a polymodal nociceptor^12^,^13^,^14^. While TRPV1 was initially cloned as a receptor for capsaicin, the pungent compound in hot chili peppers, it is known as a prototypical heat-sensing channel involved in detecting ambient environment to maintain stable body temperature in mammals and transduce heat pain^13^,^15^. Its highly temperature-sensitive activation process is accompanied by large changes in enthalpy and entropy, indicating the occurrence of a substantial conformational change^16^,^17^,^18^. However, what channel structures participate in the conformational rearrangement has been the topic of heated debates. Absence of a clear answer to this fundamental question has greatly hindered research into the heat activation mechanism that underlies temperature and pain sensation. Identification of RhTx as a TRPV1-targeting toxin provided the opportunity to address this question from a new angle. In the present study, we used a combination of animal behaviour tests, nuclear magnetic resonance (NMR), mutagenesis, electrophysiology, fluorescence imaging and Rosetta-based structural modelling to elucidate the structural and molecular basis enabling RhTx to interact with and activate TRPV1 to produce sharp pain.

## Results

### RhTx elicits pain by targeting TRPV1

**Figure 1:**
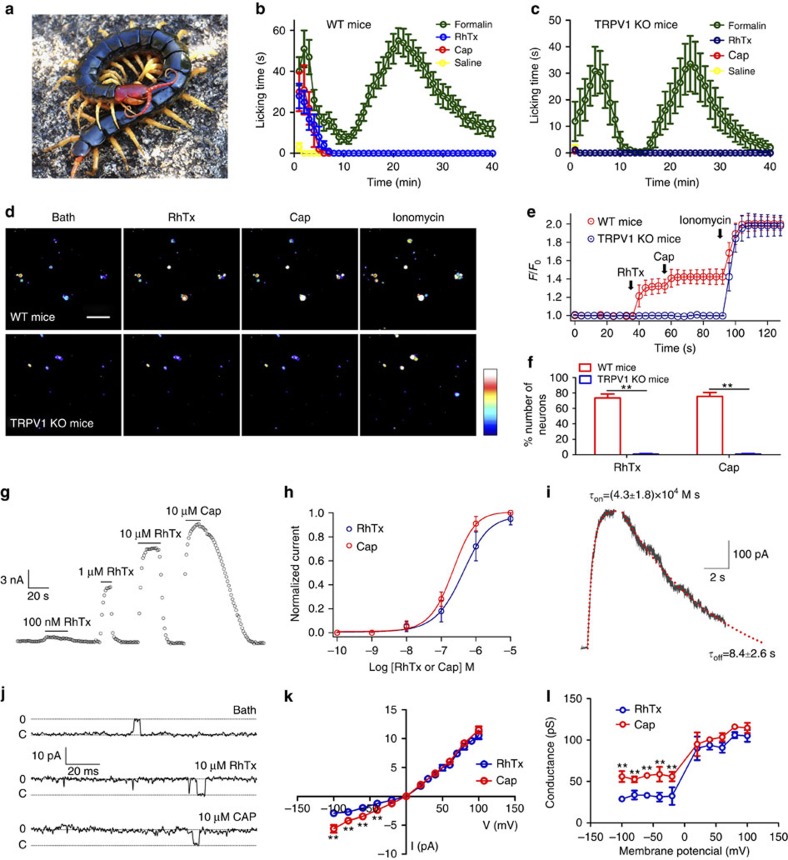
RhTx directly activates TRPV1 to induce pain. (**a**) Image of the Chinese Red-headed centipede. (**b**,**c**) Paw-licking behaviour of WT mice (**b**) or TRPV1 knockout (KO) mice (**c**) following injection of formalin, RhTx, capsaicin or saline. (**d**) Calcium imaging of DRG neurons from WT (top row) or TRPV1 KO (bottom row) mice challenged sequentially with RhTx (10 μM), capsaicin (10 μM) and ionomycin (1 mM). Scale bar, 250 μm. (**e**) Representative calcium fluorescence signals of DRG neurons from WT or TRPV1 KO mice were counted from three cells. (**f**) Positive and negative cells were counted when DRG neurons from WT or TRPV1 KO mice were challenged with RhTx (10 μM) or capsaicin (10 μM). **P<0.001, *n*=200. (**g**) Whole-cell mTRPV1 currents elicited by application of RhTx and capsaicin. (**h**) Dose–response relationships for RhTx and capsaicin overlapped with fits of a Hill equation. The effector concentration for half-maximum response and Hill slope values (average±s.e.m.) are: for RhTx, 521.5±162.1 nM, 1.17±0.37 (*n*=10); for capsaicin, 213.8±20.6 nM, 1.36±0.18 (*n*=10). (**i**) Representative time course of RhTx-induced activation and deactivation recorded from an outside-out patch at +80 mV from a holding potential of 0 mV, superimposed with fittings of a single-exponential function (red dotted curves) and mean *τ*_on_ and *τ*_off_ values (*n*=3 each). (**j**) Representative single-channel traces. (**k**,**l**) Comparison of single-channel *I*–*V* relationships (**k**) and conductances (**l**). **P<0.001 (*n*=3–9).

Our initial animal study showed that, similar to a centipede bite, raw centipede venom elicited strong pain behaviour when injected in mice (Supplementary Fig. 1). From the venom we identified one peptide, RhTx, that elicited similar pain behaviour (Fig. 1b; Supplementary Fig. 2). The pain behaviour was distinct from those mediated by inflammation but exhibited a close resemblance to that associated with capsaicin injection. When tested in *Trpv1*^*−/−*^ knockout mice, both RhTx and capsaicin were ineffective in producing the pain behaviour (Fig. 1c). We further tested RhTx on dorsal root ganglion (DRG) neurons in culture, and found that the toxin could elicit an intracellular calcium increase nearly as strong as capsaicin in all capsaicin-responsive neurons but not capsaicin-irresponsive neurons (Fig. 1d,e). These observations suggest that RhTx may target TRPV1 in sensory neurons to cause pain.

Using HEK293 cells overexpressing TRPV1, we confirmed that the channel is indeed the RhTx target. Similar to VaTx and DkTx, RhTx is an extremely potent activator for TRPV1 (Fig. 1f,g). Both efficacy and apparent binding affinity of RhTx were similar to those of capsaicin and DkTx. Kinetic analysis of RhTx-induced channel activation revealed that the high affinity (average±s.e.m.: 0.52±0.16 μM; *n*=10) is resultant from a combination of very rapid binding and slow unbinding (Fig. 1h). In comparison, DkTx achieves high affinity by compensating sluggish binding with extremely slow unbinding^5^. Therefore, RhTx is much preferable for kinetic analysis of toxin–channel interaction, while DkTx is advantageous for biochemical analysis. RhTx also exhibited high specificity for TRPV1. While it strongly activated both human and mouse TRPV1, no discernable response was observed from the homologous TRPV2–4 channels or voltage-gated K, Na and Ca channels (Supplementary Fig. 2). Single-channel analysis further confirmed high-potency activation of TRPV1 by RhTx, revealing a near unity open probability (Fig. 1i). The properties of RhTx-elicited single-channel currents closely resembled those elicited by capsaicin except for a noticeable reduction of the inward current amplitude (Fig. 1i–k), indicating that the bound toxin may interfere with ion permeation (which we will address later).

### RhTx is a small compact peptide toxin

**Figure 2:**
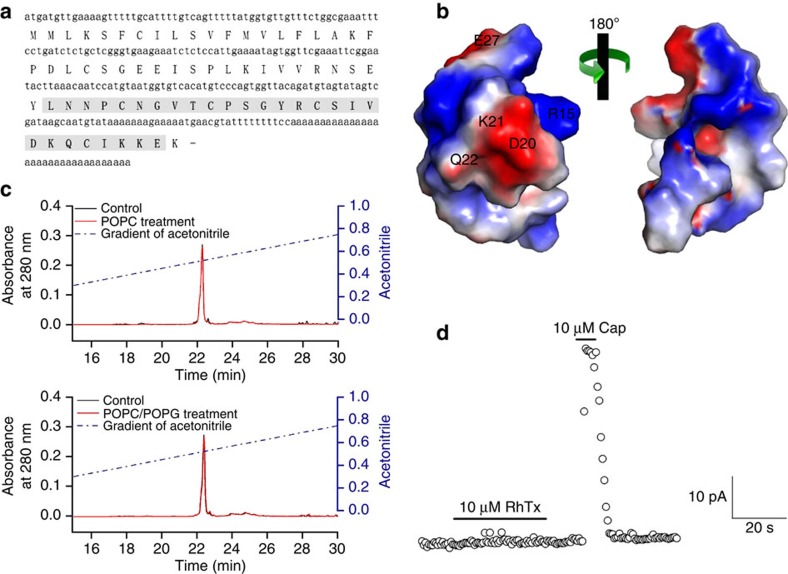
RhTx is a small polar molecule. (**a**) The cDNA and amino acid sequences of RhTx, illustrating the signal and mature peptide (shaded). (**b**) NMR structural model of RhTx, with the electrostatic potential distribution shown in colour (red=negative, blue=positive). (**c**) No difference between RP-HPLC chromatographs of 0.1 mg RhTx before (black) and after (red) treatment with POPC (top panel) or POPE/POPG (bottom panel), indicating an absence of interaction between RhTx and lipid membrane. (**d**) RhTx has no activity when exposed to the intracellular side of an inside-out patch containing mTRPV1. The holding potential was 0 mV and testing potential was +80 mV.

The gene encoding RhTx was cloned, which translates into a 69-amino-acid peptide that shows no resemblance to any known animal toxin (Fig. 2a). Comparing the gene with purified toxin indicated that the peptide undergoes substantial post-translational modification by removing more than half of its mass, yielding a mature toxin of only 27 amino acids in length. This places RhTx among the small animal peptide toxins (Supplementary Table 1). Post-translational modifications in many animal toxins help to ensure proper folding and disulfide bond formation, and to expose a protected active surface for target binding^19^. NMR spectroscopy analysis revealed that RhTx is a compact protein (Fig. 2b). Two pairs of disulfide bonds hold the folded peptide together; the N terminus is likely to be flexible (Supplementary Fig. 4). All charged residues are found in the C-terminal half of the peptide sequence. Interestingly, in the three-dimensional structure these residues are clearly visible from one side of the molecule, making RhTx a polarized molecule (Fig. 2b). While computational analysis suggested potential interaction at the lipid–solution interface (Supplementary Fig. 5), initial partition experiments indicated that RhTx does not incorporate into the lipid membrane (Fig. 2c). RhTx was completely ineffective when applied from the intracellular side (Fig. 2d). Overall, our findings suggest that RhTx likely targets a part of TRPV1 exposed to the extracellular aqueous environment. The combination of a small size, compact packing and rapid binding to an exposed channel structure underlies the instant pain onset of centipede bites.

### RhTx promotes heat-dependent activation of TRPV1

**Figure 3:**
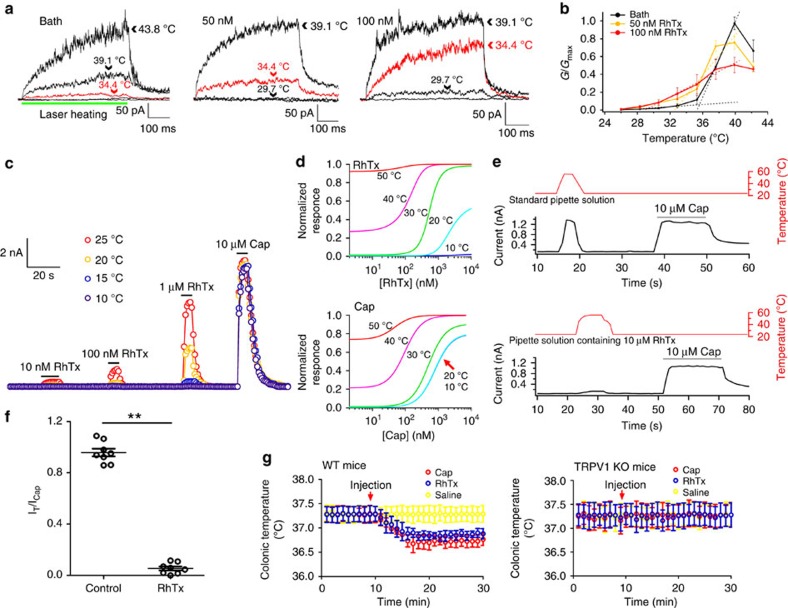
RhTx causes TRPV1 to be heat-activated. (**a**) RhTx at 50 nM (middle panel) and 100 nM (right panel) substantially potentiated infrared laser-induced heat activation. Green bar indicates the duration of laser irradiation; the temperatures reached by laser irradiation are labelled. The holding potential of these inside-out patch recordings was 0 mV and testing potential was +80 mV. (**b**) Mean (filled symbols) heat-induced responses in the presence of 50 nM or 100 nM RhTx were normalized by 10 μM capsaicin-induced currents. Dash lines represent fits to the leak current and TRPV1 channel current. (**c**) Cooling inhibited RhTx-induced channel activation but not capsaicin-induced activation. (**d**) Simulations based on a multi-allosteric gating framework (see Supplementary Fig. 6). Cooling below 20 °C could inhibit RhTx activation (top panel) but did not further inhibit capsaicin activation (bottom panel). (**e**) Incubation with 10 μM RhTx (bottom panel) selectively desensitized TRPV1 heat activation but not capsaicin activation in inside-out patches. (**f**) Summary of the ratio between current evoked by raising temperature to 55 °C and current evoked by 10 μM capsaicin in standard pipette solution and a pipette solution containing 10 μM RhTx. **P<0.001; *n*=8. (**g**) Colonic temperature of WT mice (*n*=6) or TRPV1 KO mice (*n*=3) upon injection of RhTx, capsaicin or saline.

TRPV1 is a polymodal nociceptor for which various physical and chemical stimuli facilitate each other in promoting channel activation. We found through systematic functional examinations that RhTx strongly promotes the heat activation process by down-shifting the activation threshold temperature (Fig. 3a,b). At 100 nM (5 times below effector concentration for half-maximum response), RhTx already lowered the activation threshold temperature by 6 °C, making the body temperatures of mouse and human above the activation threshold. The high temperature sensitivity exhibited by RhTx–TRPV1 interaction is in contrast to the apparently low temperature sensitivity of VaTx–TRPV1 interaction, which was suggested to resemble hanatoxin–Kv interaction^4^. We further found that lowering the experimental temperature completely prohibited channel activation induced by RhTx even at 1 μM concentration (Fig. 3c). This observation is particularly noteworthy because the same operation could not prevent capsaicin activation (Fig. 3c). It indicated that the action of RhTx requires a transition of the heat activation pathway, and cooling prevented the transition from occurring. While reciprocal potentiation is characteristic for allosterically coupled activators, selective inhibition of RhTx-induced activation by cooling could only be produced by the multi-allosteric model^20^ assuming that RhTx works through the heat activation pathway (Fig. 3d; Supplementary Fig. 6). Acting through the capsaicin-independent heat activation pathway would allow centipedes to inflict pain to their predators such as birds and snakes whose TRPV1 channels are capsaicin-insensitive^21^.

To further investigate the relationship between RhTx- and heat-induced channel activations, we compared the channel desensitization process in the absence or presence of RhTx. As described previously, TRPV1 undergoes Ca^2+^-independent slow desensitization upon extended heating, a process distinct from Ca^2+^-dependent rapid desensitization^20^. Desensitization is evident from heat-induced activations under a Ca^2+^-free condition shown in Fig. 3b. If RhTx selectively promotes TRPV1 heat activation, it is anticipated that extended RhTx treatment would also lead to channel desensitization just as heat does. When we compared extended recordings with 10 μM RhTx in the patch pipette solution to those with the standard solution, it was obvious that indeed RhTx incubation caused TRPV1 channels to be incapable to respond to heat (Fig. 3e,f). We further observed that desensitized channels remained to be responsive to capsaicin, arguing for high specificity of RhTx-induced desensitization that affected the heat activation machinery.

Many animal toxins inhibit their target through an allosteric mechanism, by preferably binding to and stabilizing the resting state of the activation machinery^22^,^23^,^24^. Our results collectively demonstrated that RhTx also controls TRPV1 activation allosterically; however, by preferably binding to the activated state, it promotes TRPV1 opening. Holding the channel closed by cooling prevented binding of RhTx, leaving the toxin ineffective (Fig. 3c,d). Cooling did not prevent capsaicin activation because it does not work through the heat activation pathway^20^. As expected, because TRPV1 is actively involved in body thermal homeostasis, RhTx-induced *in vivo* TRPV1 activation at normal body temperature led to a rapid drop in core temperature, a phenomenon that was absent in *Trpv1*^*−/−*^ mice (Fig. 3e).

### The charge-rich C terminus of RhTx mediates binding to TRPV1

**Figure 4:**
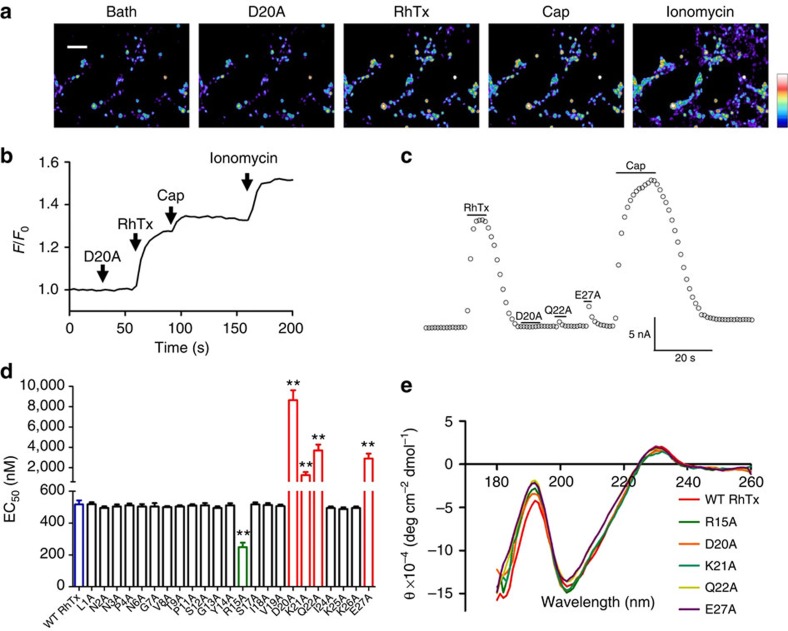
Identification of the active surface of RhTx. (**a**) Calcium imaging of mTRPV1-expressing HEK293 cells challenged by 10 μM RhTx point mutant (D20A), 10 μM RhTx, 10 μM capsaicin and 1 mM ionomycin, respectively. Scale bar, 100 μm. (**b**) Representative trace of calcium signal. (**c**) Comparison of mTRPV1 responses induced by RhTx mutants (1 μM each) to that induced by 1 μM wild-type toxin and 10 μM capsaicin. The holding potential of this whole-cell recording was 0 mV and testing potential was +80 mV. (**d**) EC_50_ values of WT RhTx and mutants. **P<0.001 (*n*=3–8). (**e**) CD spectra of WT RhTx and mutants exhibit no significant difference. EC_50_, effector concentration for half-maximum response.

Because RhTx directly targets heat activation of TRPV1, it provides a unique opportunity to identify channel structural components involved in the heat activation process. Towards this goal, we first aimed to locate the channel-binding surface of RhTx. We synthesized mutant toxins that contained an alanine at each of the 23 non-cysteine positions. Among them, four mutants (D20A, K21A, Q22A and E27A) lost most but not all of the agonist activity (Fig. 4), which was found to be due to a marked reduction in the apparent binding affinity (Fig. 4d). Another mutation (R15A) enhanced the apparent binding affinity (Fig. 4d). In agreement with the presence of remaining agonist activity, all mutants exhibited wild-type (WT)-like structural features (Fig. 4e), suggesting that the deterioration in agonist activity was due to weakened binding. Intriguingly, four out of the five identified residues are charged, whereas the fifth one is polar. They are distributed on the same face of the toxin (Fig. 2b). The absence of a detectable effect by mutating hydrophobic residues is consistent with the finding that RhTx did not incorporate into lipid (Fig. 2c). We conclude that the charged surface of RhTx mediates binding to TRPV1. Consistent with this conclusion, toxins carrying a fluorescein isothiocyanate moiety at its flexible N terminus retained agonist activity (Supplementary Fig. 7).

### RhTx binds to the outer pore region of TRPV1

**Figure 5:**
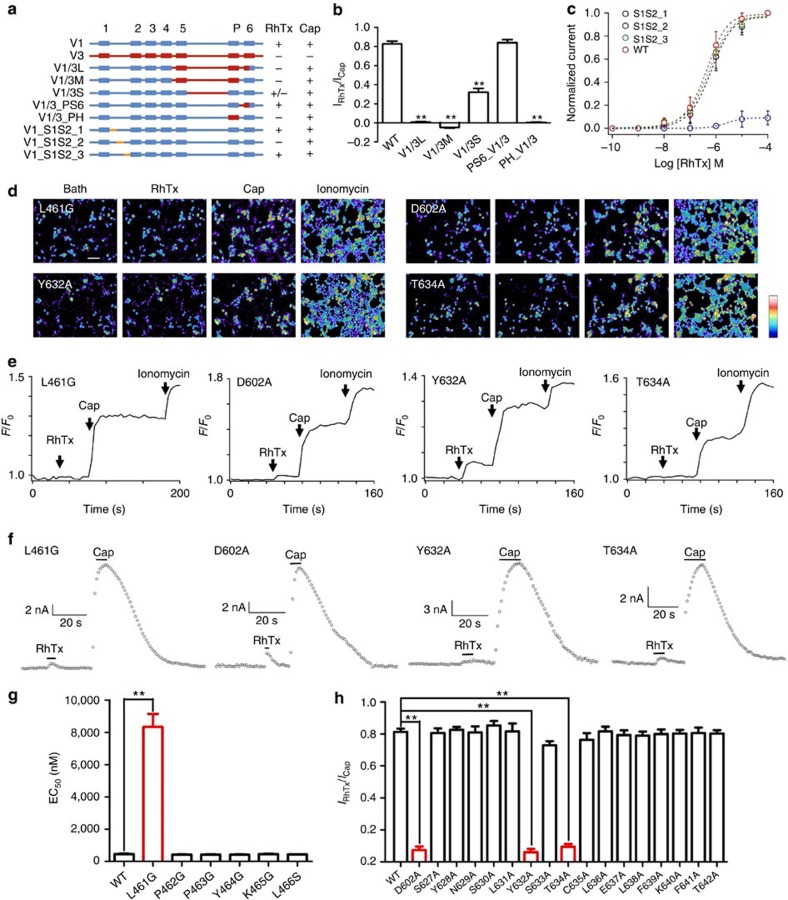
RhTx targets the TRPV1 outer pore. (**a**) Responsiveness to RhTx and capsaicin by chimeric channels between mTRPV1 (blue) and mTRPV3 (red) or TRPV1 mutants containing a GGGGS sequence replacement (yellow). (**b**) Comparison of changes in RhTx sensitivity. **P<0.001 (*n*=4–6). (**c**) Dose–response relationships of WT and mutant channels containing a GGGGS replacement in the S1–S2 linker. (**d**,**e**) Calcium imaging (**d**) and average fluorescence response (**e**) of mTRPV1 point mutants to 10 μM RhTx, 10 μM capsaicin and 1 μM ionomycin, respectively. Scale bars, 100 μm (horizontal), 300 to 2000 AU (vertical). (**f**) Representative whole-cell current response of point mutants to RhTx and capsaicin (both at 10 μM). The holding potential was 0 mV and testing potential was +80 mV. (**g**) Comparison of EC_50_ values of WT and point mutants at the S1–S2 linker. (**h**) Comparison of RhTx responses of WT and point mutants at the outer pore. EC_50_, effector concentration for half-maximum response.

We next searched for the toxin-binding site on TRPV1. A series of chimeras were made between TRPV1 and the toxin- and capsaicin-insensitive TRPV3 (Fig. 5a; Supplementary Fig. 8). Among them, chimeras containing the pore region of TRPV3 exhibited disrupted toxin sensitivity, while they remained capsaicin-sensitive because the capsaicin-binding site^21^,^25^,^26^ was intact (Fig. 5a,b). Specifically, replacing the pore helix or turret had a major impact on toxin sensitivity, while replacing the ion selectivity filter and its posterior loop did not have an obvious effect. These results suggest that RhTx binds to the charge-rich outer pore region where it may directly interact with the pore helix and turret, two adjacent structural elements known to be critical for activation gating of TRPV1 (refs 17, 27, 28, 29). Interestingly, DkTx is known to bind to the same general region of TRPV1 (refs 5, 25), suggesting that the two peptide toxins may target the same gating machinery.

Beyond the outer pore region, we found that the S1–S2 linker also contributes to toxin sensitivity. Using a series of sequence-replacement mutants^29^, we found the middle part of this extracellular loop necessary for RhTx to exhibit agonist activity (Fig. 5a,c). It is possible that a part of the bound RhTx may reach outward to this peripheral location. However, given the size of the toxin molecule, it is also possible that interaction between the S1–S2 linker and the pore domain may be required for normal activation gating^29^.

### Molecular interactions between RhTx and TRPV1

To understand the molecular interactions between RhTx and TRPV1, we first conducted a point-mutation screening. Functional examination of these mutations identified D602 in the turret, Y632 and T634 in the pore helix and L461 in the S1–S2 linker to be critical for RhTx-induced channel activation (Fig. 5d–h). A glycine or alanine mutation at these key positions markedly reduced both toxin-induced intracellular calcium increase (Fig. 5d,e) and current (Fig. 5f–h) while again sparing capsaicin activation. Since the mutant channels also exhibited near normal responses to H^+^, Mg^2+^ and 2-APB (Supplementary Fig. 9), these point mutations affected mostly RhTx–TRPV1 interaction.

**Figure 6:**
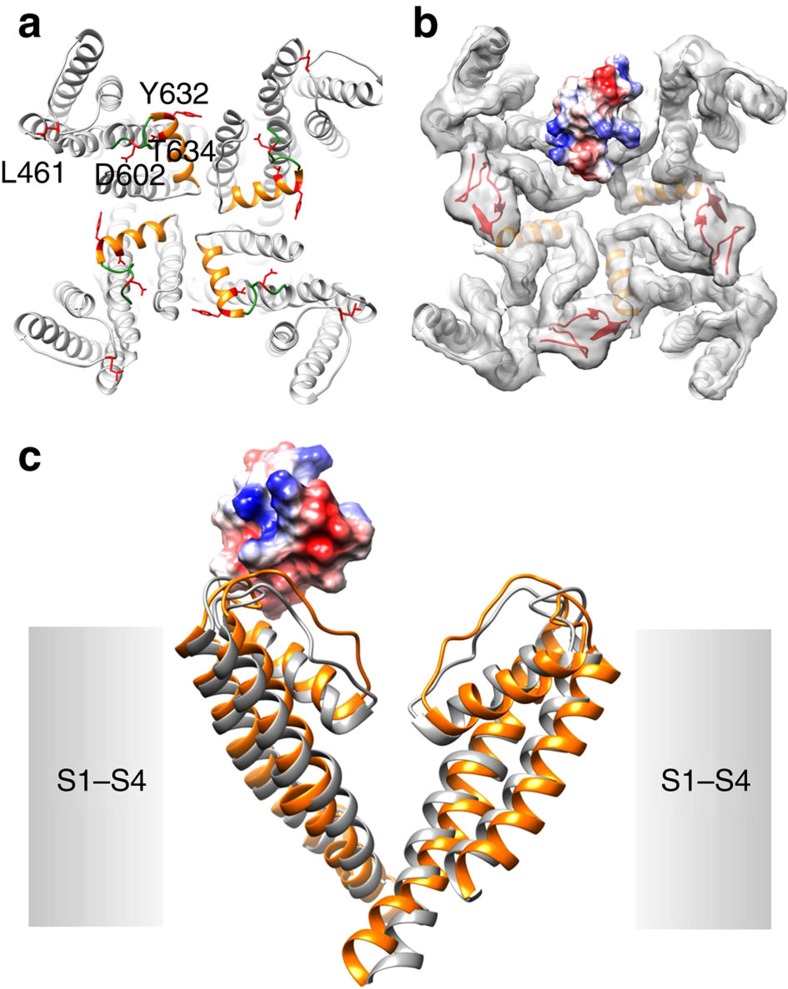
RhTx binds to the outer pore region of TRPV1. (**a**) Location of key residues identified by mutagenesis (with the side chain shown in red) mapped to the cryo-EM structure (closed state). Turret and pore helix are shown in orange and green, respectively. (**b**) Molecular docking of RhTx (coloured by surface electrostatic potential: red, negatively charged; blue, positively charged) to TRPV1, using the DkTx-bound state as the starting template. The backbone of TRPV1 and DkTx are shown in grey and red, respectively, with the electron density map superimposed. (**c**) Comparison of channel conformations between the closed state (grey) and RhTx-bound open state (orange).

When mapping identified residues onto the cryo-EM structure of TRPV1 (refs 25, 30), it became clear that D602, Y632 and T634 are clustered at the rim of the outer pore (Fig. 6a). The observation, in close agreement with results from chimera tests, provided constrains for the location of bound RhTx and sites participating in toxin–channel interactions. Utilizing this crucial information and the available TRPV1 cryo-EM structures^25^,^30^, we used Rosetta-based molecular docking to determine the position of RhTx in complex with TRPV1. Our results suggest that the bound RhTx molecule is wedged into the extracellular crevice formed between neighbouring subunits, where it may directly interact with turret and pore helix through electrostatic and hydrophobic interactions (Fig. 6b; Supplementary Movie). At this position, the toxin may also affect the entry of permeable ions into the pore, explaining its permeation effect (Fig. 1j,k). On the basis of these results, binding of RhTx is expected to induce conformational rearrangement to the outer pore to cause heat activation of the channel (Fig. 6c).

## Discussion

Our study identified a novel small peptide toxin that strongly activates TRPV1 with rapid kinetics, opening up new opportunities to investigate TRPV1 activation mechanism. Identification that RhTx targets the outer pore to cause TRPV1 heat activation is particularly interesting from the mechanistic point of view. The outer pore is a known hot spot for mediating the action of many chemical activators such as H^+^ (refs 31, 32), divalent cations^29^,^33^ and DkTx^5^. In addition, many mutations at this region left the mutant channel activated at room temperature in the absence of chemical activator^17^,^27^,^28^,^29^ or prevented heat activation^34^. While possible involvement of heat activation has been suggested for these mutational effects, demonstration of RhTx-induced heat activation provided direct evidence in WT channels that conformational change of the outer pore can induce heat activation. Similar to RhTx, divalent cations were previously found to potentiate TRPV1 heat activation by affecting extracellular channel structures^20^,^29^, although the exact site(s) of interaction was unclear. In support of the view that RhTx and divalent cations interact with TRPV1 outer pore to promote heat activation, fluorophore tags on the pore turret have previously revealed that heat activation (but not capsaicin activation) requires direct participation of the outer pore region^17^,^29^.

How do conformational changes induced by binding of RhTx open the channel? It remains unclear how TRPV1 and related channel senses heat. The present study does not necessarily suggest that RhTx affects the ‘heat sensor’ of the channel. From an allosteric view, the RhTx-bound outer pore conformation favours the open state of the channel, indicating that the outer pore is an integrated part of the heat activation machinery. The emerging picture from both cryo-EM data and functional studies is that in TRPV1 the peripheral S1–S4 domains form a rigid supportive structure cradling the dynamic central pore domain (reviewed by Zheng and Ma^35^). Given the close proximity of the outer pore to the ion selectivity filter, it is conceivable that conformational change in the outer pore caused by RhTx binding may directly influence the upper gate indicated by the cryo-EM structures^25^,^30^. Gating of the selectivity filter is observed during activation of cyclic nucleotide-gated channels^36^,^37^, BK channels^38^, Shaker channels^39^ and KcsA channels^40^. A dynamic ion selectivity filter in TRPV1 is recently suggested^41^. In contrast, channel activation by capsaicin and intracellular modulators including PIP2 and calmodulin are mediated by a separate set of channel structures that appear to converge to the bottom of the pore domain^26^, which must be functionally coupled with the selectivity filter gate.

Being small predators, centipedes pack in their venoms distinct toxins evolved specifically for hunting and self-defence^6^,^7^. Some toxins are lethal to insects and worms that form their major food supply, by inhibiting Na_V_ channels^42^. Others inhibit vertebrate K_V_ channels to cause hyper-excitability^43^. Through evolution, centipede has also found a way to highjack the gating machinery of TRPV1 reserved for heat (and perhaps other extracellular activators) to inflict intense burning pain. RhTx adopts a different channel-binding strategy from the structurally distinct DkTx, a 75-amino-acid-long peptide toxin. DkTx binds TRPV1 slowly but reaches high affinity due to antibody-like binding of two active knots^5^,^25^. RhTx binds and unbinds much more rapidly but achieves a comparable affinity to DkTx and higher than that of an isolated DkTx knot. These properties make RhTx an especially powerful tool for future investigation to functionally dissect the heat activation mechanism of TRPV1.

As an attractive target for pain medication, TRPV1 is currently been investigated as an entrance portal for pain-killer QX-314 or cytotoxic Ca^2+^ to inhibit sensory neurons^44^,^45^. Peptide toxins such as conotoxins have shown great promises as a new type of pain medicine^46^. While RhTx is an activator of TRPV1, knowing how it interacts with TRPV1 protein opens the door for molecular modification of the toxin peptide to alter and even reverse its activation properties. Therefore, RhTx and its derivatives, being synthesizable small peptide agonists, may open a novel path to directly control the activity of nociceptors.

## Methods

### Neurotoxin purification and protein sequencing

Adult *Scolopendra subspinipes mutilans* L. Koch (both sexes, *n*=1,000) were purchased from Jiangsu Province, China. As previously reported^7^, venom was collected manually by stimulating the venom glands with a 3-V alternating current. The unique peptide toxin was purified from the raw venom using a combination of a Sephadex G-50 gel filtration column and reverse-phase (RP)-HPLC (Supplementary Fig. 3). The purity of toxin was analysed using a matrix-assisted laser desorption ionization time-of-flight (MALDI-TOF). Lyophilized HPLC fractions were dissolved in 0.1% (v/v) trifluoroacetic acid/water, from which 0.5 μl was spotted onto a MALDI-TOF plate with 0.5 μl α-cyano-4-hydroxycinnamic acid matrix (10 mg ml^−1^ in 60% acetonitrile). Spots were analysed by an UltraFlex I mass spectrometer (Bruker Daltonics) in a positive ion mode. The toxin with purity over 99.5% was collected and stored at −20 °C until further use. A Shimadzu protein sequencer (PPSQ-31A, Shimadzu, Japan) was used for the determination of primary sequence of toxin.

### cDNA library and cloning

The venom-gland complementary DNA (cDNA) library was prepared as previously described^47^. Briefly, the total RNAs were extracted from the venom glands of 20 centipedes using TRIzol (Life Technologies Ltd). This was used to prepare the cDNA library using a SMART PCR cDNA synthesis kit (Clontech, Palo Alto, CA). The first strand was synthesized using the 3′ SMART CDS Primer II A (5′-AAGCAGTGGTATCAACGCAGAGT ACT(30)N_−1_N-3′, where N=A, C, G or T and N_−1_=A, G or C) and SMART II A oligonucleotide, (5′-AAGCAGTGGTATCAACGCAGAGTACGCGGG-3′). The 5′ PCR primer II A (5′-AAGCAGTGGTATCAACGCAGAGT-3′) provided by the kit was used to synthesize the second strand using Advantage polymerase (Clontech). RACE (Rapid Amplification of cDNA ends) was used to clone transcripts encoding RhTx from the venom-gland cDNA library.

For cloning, sense-direction primers were designed according to the amino-acid sequences determined by Edman degradation. These primers (5′-ATGATGYTNAARWSNTTYTGY-3′, 5′-CGTTTTTGAAAAGTTGTAGTA-3′) were used in conjunction with an antisense SMART II A primer II in PCR reactions to screen for transcripts encoding neurotoxins. PCR was performed using Advantage polymerase (Clontech) under the following conditions: 2 min at 94 °C, followed by 30 cycles of 10 s at 92 °C, 30 s at 50 °C and 40 s at 72 °C. Finally, the PCR products were cloned into pGEM-T Easy vector (Promega, Madison, WI). DNA sequencing was performed on an ABI PRISM 377 DNA sequencer (Applied Biosystems).

### Determination of disulfide bridge connections

RhTx (0.1 mg) was partially reduced in 10 μl of citrate buffer (1 M, pH 3.0) containing 6 M guanidine-HCl and 0.05 M Tris (2-carboxyethyl)phosphine for 10 min at 40 °C. The partially reduced sample was fractionated by C18 RP-HPLC using a linear acetonitrile gradient (0–60% over 60 min) (Supplementary Fig. 4). The fractions of intermediates with free thiols were collected and determined using MALDI-TOF mass spectrometry. Reduced RhTx with two free thiols were lyophilized and alkylated with iodoacetamide (0.5 M, pH 8.3). Alkylated peptides were purified, desalted using C18 RP-HPLC and subjected to Edman degradation on a PPSQ-31A Shimadzu protein sequencer.

### NMR data acquisition and structure determination

The RhTx sample for NMR measurement contained 4 mmol l^−1^ peptides in 500 μl of 90% PBS/10% D_2_O at pH 6.5. All NMR experiments were carried out on a 600-MHz Bruker AV600 spectrometer equipped with three RF channels. The two-dimensional TOCSY spectra were acquired with a mixing time of 75 ms. NOESY spectra were acquired with mixing times of 100, 200 and 300 ms. Both the Watergate approach and the pre-saturation scheme were employed for water suppression. All spectra were recorded with 400 t1 increments and 2,048 complex data points. Signals were averaged over 32 transients. All NMR data were processed and analysed using the NMRPipe/NMRDraw software and the Sparky program^48^,^49^. Linear prediction in the t1 dimension was used before the Fourier transformation. ^1^H resonance assignments were performed using TOCSY, NOESY and COSY spectra for identification of the scalar coupled spin systems and the sequential connectivity.

^1^H–^1^H distance restraints were derived primarily from the NOESY spectra recorded in PBS with a mixing time of 300 ms. Structure calculations were performed according to the standard ARIA/CNS protocol^50^,^51^,^52^. NOE distance constraints are shown in Supplementary Table 2. A family of 200 structures was calculated according to the simulated annealing protocol and the 10 lowest-energy structures were finally selected. The root mean squared deviation (r.m.s.d.) values for the backbone atoms of secondary structural regions were 0.75 Å, calculated by the program MOLMOL^53^. Ramachandran plot analysis was performed using the PROCHECK program. The electrostatic potential graph was displayed by the software PyMol.

### Toxin peptides synthesis and purification

Synthesis of linear RhTx and point mutations were carried out on an automatic peptide synthesizer (PerSeptive Biosystems) using an Fmoc/tert-butyl strategy and HOBt/TBTU/NMM coupling method. Crude reduced peptides were purified by RP-HPLC. Once the purity of a peptide of interest was determined to be higher than 95% by MALDI-TOF mass spectrometry and HPLC techniques, the peak was pooled and lyophilized. The linear reduced peptide was dissolved in 0.1 M Tris-HCl buffer (pH 8.0) at a final concentration of 30 μM glutathione containing 5 mM reduced glutathione and 0.5 mM oxidized glutathione. Oxidization and folding were performed at room temperature and monitored at 280 nm by analytical RP-HPLC and MALDI-TOF mass spectrometry.

### Circular dichroism spectroscopy

Circular dichroism (CD) spectroscopy was performed using a Jasco J-715 spectrophotometer (Jasco). The secondary and tertiary structures of purified RhTx were determined by obtaining CD spectra at far-ultraviolet (250–190 nm) and near-ultraviolet (350–250 nm), respectively. Far-ultraviolet CD spectra were obtained in 0.1-mm path-length circular cuvettes, while near-ultraviolet spectra were sampled in 10-mm path-length standard quartz cuvettes. All data were collected using a step resolution of 1 nm, a scan speed of 50 nm min^−1^ and a response time of 1 s. Measurements were performed over 10 accumulations to reduce the signal-to-noise ratio and were baseline corrected against the storage buffer. Protein concentrations were approximately 1.0–1.5 mg ml^−1^; CD measurements were converted to units of molar ellipticity ([*θ*]). All corrections and processing were undertaken using the Jasco Standard Analysis Program.

### Small unilamellar vesicle-binding assay

Briefly, for POPC (1-palmitoyl-2-oleoyl-sn-glycero-3-phosphatidylcholine) vesicle preparation, POPC evaporated under a stream of nitrogen gas until a lipid film was observed at the bottom of the test tube. After the film rinsed with pentane was lyophilized at −40 °C overnight, the lipid was suspended with a buffer (10 mM HEPES, 50 mM KCl at pH 7.0). POPE (1-palmitoyl-2-oleoyl-phosphatidylethanolamine, 10 mg ml^−1^) and POPG (1-palmitoyl-2-oleoylphosphatidyl-Dl-glycerol, 10 mg ml^−1^) were mixed in a 3:1 ratio for POPE/POPG vesicle preparation. In total, 150 mM KCl was used instead of 50 mM KCl. The suspended lipid was then sonicated (in ice water) until the solution became transparent. RhTx was mixed with 10 mg ml^−1^ (total concentration) small unilamellar vesicle (SUVs). The mixture was incubated for 40 min at room temperature before ultracentrifugation. Toxin–vesicle mixtures were centrifuged at 304,000*g* for 150 min. The residual toxins in the supernatant were evaluated by RP-HPLC using a linear gradient of acetonitrile containing 0.1% v/v trifluoroacetic acid.

### Mutagenesis and transient transfection

TRPV1 and TRPV3 chimeras used in this study were generated by the overlapping extension method^54^ and confirmed by DNA sequencing, as described previously^29^. Briefly, to generate V1/3S, the primer pairs (5′-GTCCTTCTTGTCCTTTGAGCACTTCTCGATCAGTGTCACTACGGC-3′and 5′-CAAAGGACAAGAAGGACTGCAGTTCTTACAACAGCCTGTATTCCACAT-3′) were used. To generate other TRPV1/TRPV3 chimeras, the enzyme cutting sites by MluI were made by the primer pair (5′-ACATGCTCTACTACACGCGTGGATTCCAGCAGATGG-3′ and 5′-CCATCTGCTGGAATCCACGCGTGTAGTAGAGCATGT-3′) for TRPV1 and the primer pair (5′-ACATGCTCTACTACACGCGTGGCTTCCAGTCTATGGG-3′ and 5′-CCCATAGACTGGAAGCCACGCGTGTAGTAGAGCATGT-3′) for TRPV3. The enzyme cutting sites by PvuI were made by the primer pair (5′-GCTGTTCAAGTTCACGATCGGCATGGGTGAC-3′ and 5′-GTCACCCATGCCGATCGTGAACTTGAACAGC-3′) for TRPV1 and the primer pair (5′-CTCTTCAAGCTCACGATCGGCCTGGGCGACCT-3′ and 5′-AGGTCGCCCAGGCCGATCGTGAGCTTGAAGAG-3′) for TRPV3. The enzyme cutting sites by BsiWI were made by the primer pair (5′-CATCATCCTGTTACTGGCGTACGTGATTCTCACCTACATC-3′ and 5′-GATGTAGGTGAGAATCACGTACGCCAGTAACAGGATGATG-3′) for TRPV1 and the primer pair (5′-TCTCTTCCTACTCATCACGTACGTCATCCTCACCTTCGTC-3′ and 5′-GACGAAGGTGAGGATGACGTACGTGATGAGTAGGAAGAGA-3′) for TRPV3. V1/3M were generated by enzyme cut and paste between MluI and PvuI sites, while V1/3L were generated by enzyme cut and paste between MluI and BsiWI sites. V1_S1S2_1, V1_S1S2_3 and V1_S1S2_3 were generated as previously reported^29^. All mTRPV1 point mutations were constructed using the QuickChange II XL site-directed mutagenesis kit following the manufacturer’s instruction. These point mutations were sequenced to confirm that appropriate constructs were made. HEK-293T cells were cultured and transiently transfected using Lipofectamine 2,000 (Invitrogen) as previously described^42^.

### DRG neuron imaging and electrophysiology

Mouse DRG neurons were acutely dissociated and maintained in a short-term primary culture according to procedures as previous descripted^42^. DRG neurons or HEK-293T cells were loaded with Fluo-4 AM in 2 mM Ca^2+^ Ringer’s solution (140 mM NaCl, 5 mM KCl, 2 mM MgCl_2_, 10 mM glucose, 2 mM CaCl_2_ and 10 mM HEPES, pH 7.4). Fluorescence images of DRG neurons and HEK-293T cells were acquired with an Olympus IX81 microscope with Hamamatsu C4742 charge-coupled device camera controlled by the MetaFluor Software (Molecular Devices). Fluo-4 was excited by a mercury vapour light source with a 500/20-nm excitation filter, while fluorescence emission was detected with a 535/30-nm emission filter. Fluorescence images were required with automated routines written in MetaMorph software (Molecular Devices) and analysed with Igor Pro (Wavemetrics).

As described in details in a previous report^20^, macroscopic currents (in whole-cell, inside-out or outside-out configuration) as well as single-channel currents were recorded using a HEKA EPC10 amplifier with the PatchMaster software (HEKA). Both pipette solution and bath solution contained 130 mM NaCl, 3 mM HEPES and 0.2 mM EDTA (pH 7.2). All recordings were performed at room temperature unless otherwise stated. A holding potential of 0 mV was used from which a testing pulse to +80 mV was applied. Statistical difference was determined using the Student’s *t*-test, and indicated by ** when reaching the significance level *P*<0.001.

### Heating experiments by laser or preheated bathing solution

The experimental set-up for rapidly heating cell membrane containing expressed TRPV1 channels was described in a previous report^55^. Electrophysiological recordings were done in the inside-out mode. Toxin was added in the pipette solution. Laser light of 1,443 nm was generated by a laser diode (Fitel) driven by a controller (Thorlabs). The maximal optic output power of the laser was 250 mW. The laser light was transmitted to the voltage-clamped cell membrane through a single-mode optical fibre (125 μm outer diameter, 10 μm light-conducting core diameter), with the patch pipette tip placed in front of the centre of the optical fibre. Light energy was absorbed by water and converted to heat to drive channel activation. To calibrate the laser heating system, the relationship between laser driving power and temperature was tabulated as previously described^56^. Briefly, a glass pipette was filled with a solution distinct from the bath solution and was centred at the end of the optical fibre. The laser driving power was adjusted to produce junction potential values matching those measured in the same solutions at different temperatures.

For recording the RhTx-induced desensitization of TRPV1, temperature control was achieved by perfusion of preheated or room-temperature bathing solution. Hot bathing solution were maintained at expected temperature with an SH-27B in-line solution heater controlled by a TC-324C temperature controller (Warner). A solution exchanger RSC-200 with eight separate tubes to deliver room-temperature bathing solution and capsaicin. A TA-29 miniature bead thermistor (Harvard Apparatus) was placed right next to the pipette to ensure accurate monitoring of local temperature.

### Molecular docking of RhTx by Rosetta

To prepare structures of RhTx (PDB ID: 2MVA) and rTRPV1 channel (toxin-bounded state, PDB ID: 3J5Q) for molecular docking, they were first relaxed in Rosetta 3.4 (ref. 57). For each structure 10,000 models were generated. The top 10 lowest-energy models converged well and the lowest-energy model was chosen for docking. To dock the toxin, membrane environment was first set up on the channel model using RosettaScripts^58^,^59^. A total of 20,000 docking models were generated, from which the top 1,000 total energy score models were identified. From this pool, the top 10 models with largest binding energy were structurally converged well with Cα r.m.s.d.<2.5 Å. Among the top 10 models, the one that was in agreement with experimental data was chosen as the final RhTx–rTRPV1 docking model.

### Allosteric modelling

Potential outcomes from RhTx interacting with different TRPV1 activation machineries were predicted using a multi-allosteric gating framework^20^ (Supplementary Fig. 6). In this gating pyramid the C↔O transition at the apex, representing closed-to-open transition of the channel pore, is controlled by distinct transitions that are in turn dictated by capsaicin, voltage and heat. For simplicity, no direct interaction among activator-induced transitions was assumed, for example, *J*_CV_=*J*_CH_=*J*_VH_=1. These and all other parameters were directly taken from our recently reported study^20^, except that *J*_C_=*J*_H_=800. The effect of RhTx on channel activation was tested assuming it affects one of the activator-induced transitions (C, D or E), or through an additional branch linking to the C↔O transition, an approach described in the recent study^20^. To predict the channel’s response to capsaicin at varying temperature, the N↔P transition was calculated by setting the temperature to be at a distinct level from 10 to 50 °C while fixing the R↔A transition. The open probability was calculated as a function of capsaicin concentration. To predict the channel’s response to RhTx at varying temperature, the N↔P transition was calculated as a function of RhTx concentration at distinct temperature level from 10 to 50 °C while fixing the U↔L and R↔A transitions. The open probability under these conditions was calculated accordingly.

### Animal assays

*In vivo* effects of RhTx on WT mice or *Trpv1*^*−/−*^ mice were examined according to protocols described previously^42^. For the paw-licking assay, pain was induced in mice by intraplantar injection of 0.1% formalin, capsaicin (1 μmol per paw) or RhTx (1 μmol per paw). Each of the testing materials was dissolved in 100 μl saline. Control mice received the same volume of saline. Injected mice were placed individually into open polyvinyl cages (20 × 40 × 15 cm). Time spent licking the injected paw was recorded by a digital video camera during the following 40 min. For recording colonic temperature from WT or *Trpv1*^*−/−*^ mice, mice equipped with a copper-constantan thermocouple were individually placed in open polyvinyl cages. A data logger was connected to the thermocouple. Mice were injected intraperitoneally with 100 μl saline containing capsaicin or RhTx. The dose of capsaicin or RhTx was 10 μmol kg^−1^ for these experiments. Mice in the control group received the same volume of saline.

## Additional information

**Accession codes:** RhTx cDNA and protein sequence have been deposited in Genbank under accession number KM675476. RhTx three-dimensional structure has been deposited in RCSB PDB under accession number 2MVA.

**How to cite this article:** Yang, S. *et al*. A pain-inducing centipede toxin targets the heat activation machinery of nociceptor TRPV1. *Nat. Commun.* 6:8297 doi: 10.1038/ncomms9297 (2015).

## Supplementary Material

Supplementary Information Supplementary Figures 1-9, Supplementary Tables 1-2 and Supplementary References

Supplementary Movie RhTx docked onto the outer pore region of TRPV1. The movie depicts the RhTx-TRPV1 complex in side view (first half of the movie) and top view (second half). The backbone and electron density map of TRPV1 are shown in grey, with the pore helix highlighted in yellow; RhTx is shown with surface electrostatic potential (red = negative, blue = positive); the backbone (in red) and electron density map (in grey) of DkTx are shown as comparison.
