# Supplementary material for: A pain-inducing centipede toxin targets the heat activation machinery of nociceptor TRPV1
Source: Nat Commun. 2015 Sep 30;6:8297. doi: 10.1038/ncomms9297 (PMC4589873; doi:10.1038/ncomms9297)
Supplement: Supplementary Information — Supplementary Figures 1-9, Supplementary Tables 1-2 and Supplementary References [file ncomms9297-s1.pdf]

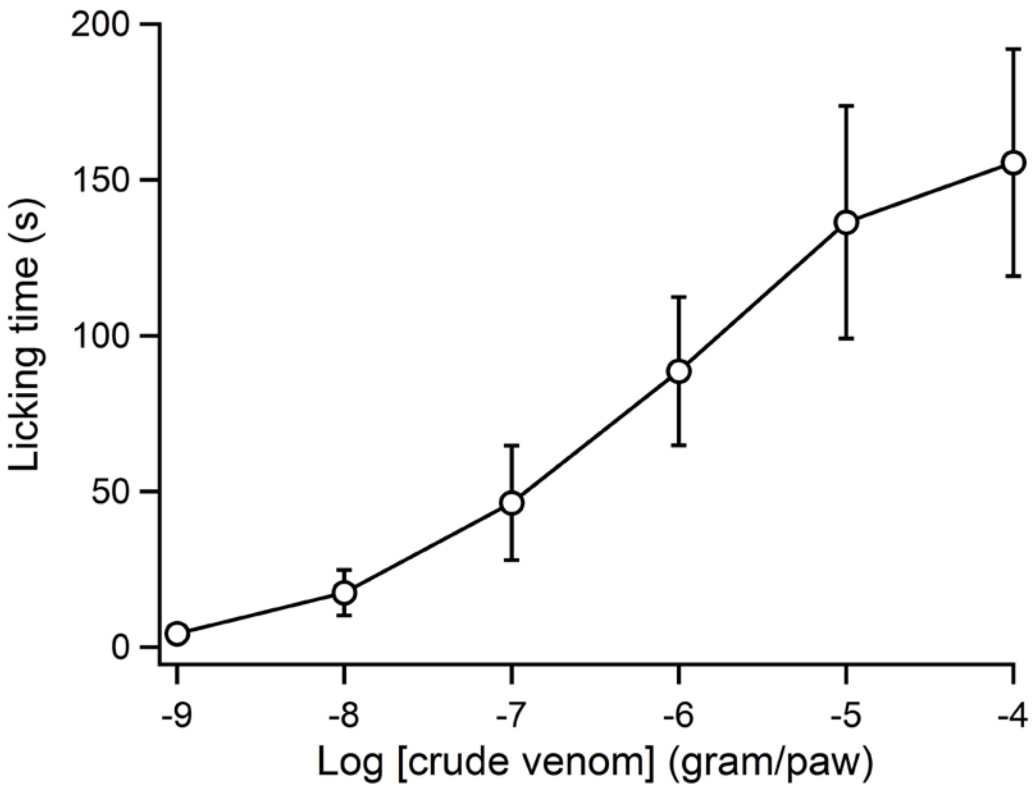

Supplementary Figure 1. Centipede crude venom-induced paw licking. Pain behavior was induced in mice by intraplantar injection of centipede crude venom. After crude venom injection, mice were immediately placed individually into open polyvinyl cages (20 × 40 × 15 cm). The time spent licking the injected paw was recorded by digital video camera during 0-10 minutes post-injection. Centipede crude venom induced strong pain behavior in a dose-dependent manner. Data points were presented as average ± sem (n = 5-to-8 in each group).

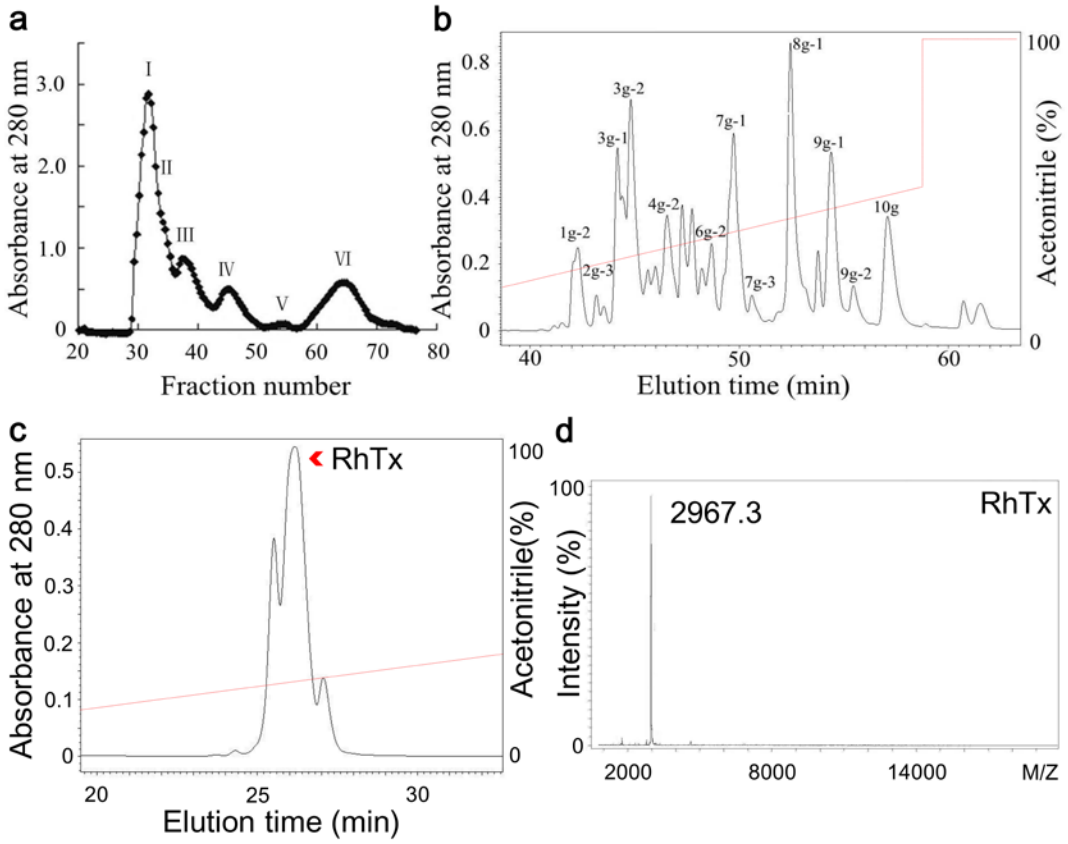

Supplementary Figure 2. Purification of RhTx. (a) Diluted crude venom of Chinese Red-headed centipede was subjected to Sephadex G-50 gel filtration. Protein fraction IV was pooled and further purified by using C18 RP-HPLC column. (b) Protein fraction 3g-1 containing RhTx was pooled. (c) RhTx was purified using analytical C18 RP-HPLC. (d) MALDI-TOF spectral analysis of RhTx revealed its molecular weight at 2967.3 Da.

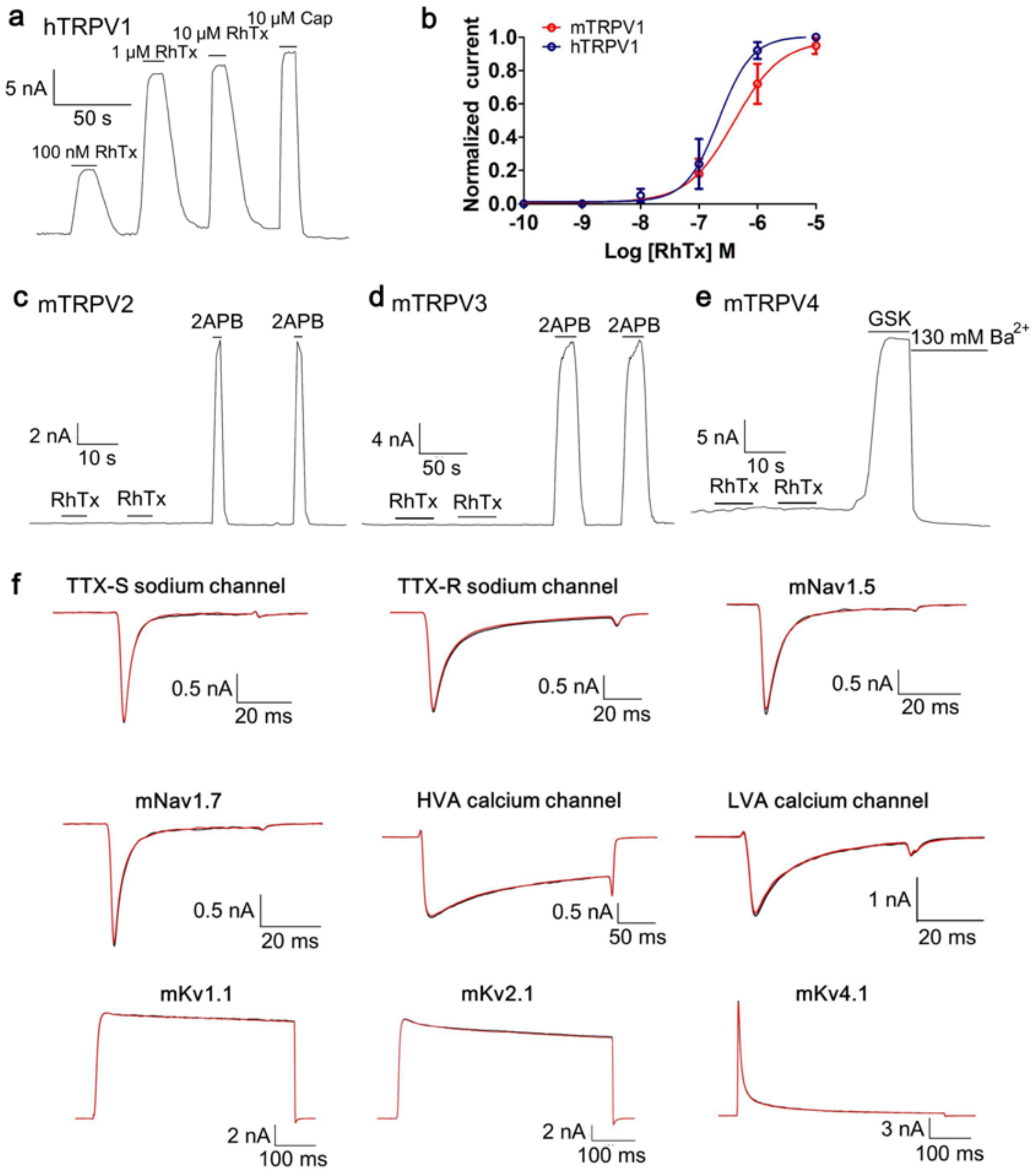

Supplementary Figure 3. RhTx is highly selective for TRPV1. (a) A representative whole-cell recording of hTRPV1 treated with RhTx or capsaicin. (b) Comparison of the dose-response relationships between hTRPV1 and mTRPV1. (c-e) RhTx at 10 μM has no detectable effect on mTRPV2 (c), mTRPV3 (d) and mTRPV4 (e). (f) Treatment with 10 μM RhTx (red traces) exhibited no detectable effect compared to toxin-free treatment (black traces) on voltage-gated Na, K, Ca channels.

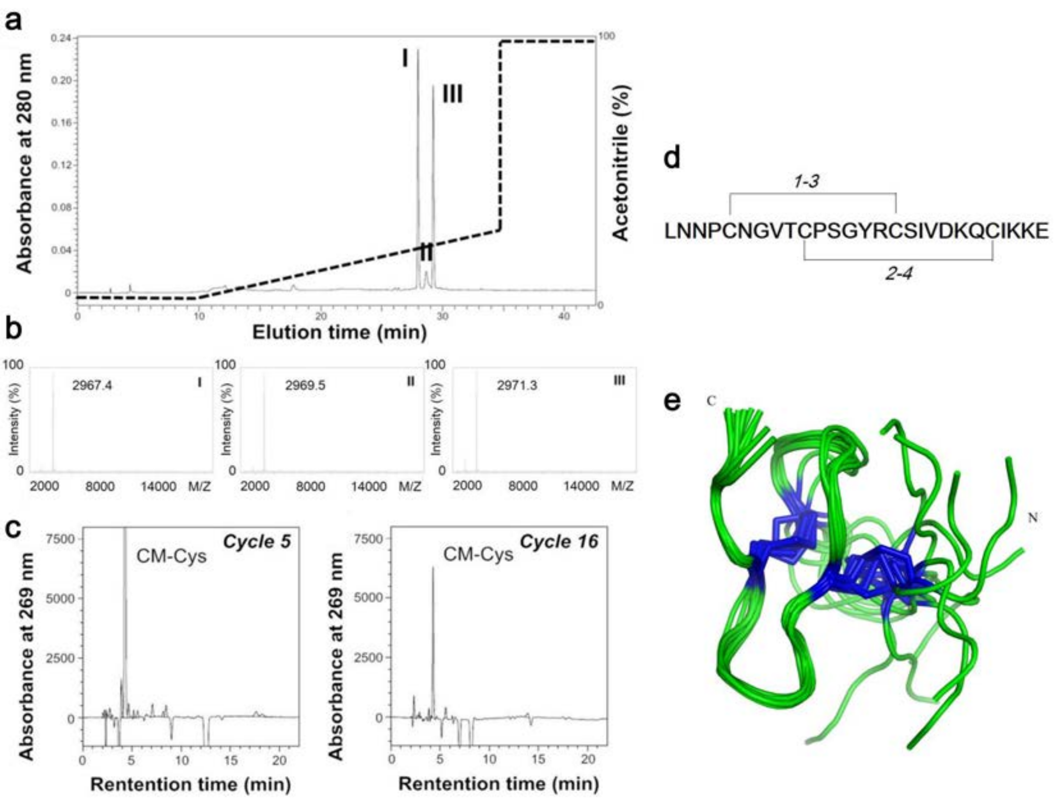

Supplementary Figure 4. Determination of disulfide bridges in RhTx and NMR structures. (a) RP-HPLC chromatograph of RhTx after partial reduction with TCEP, which yielded three major species labeled as I, II, and III. (b-d) MALDI-TOF spectral analysis of peak I, II and III indicated that they correspond to non-reduced RhTx (peak I), RhTx with one reduced disulfide bridge (peak II), or RhTx with two reduced disulfide bridges (peak III). Partially reduced peptides of peak II were alkylated with iodoacetamide for 1 min and then purified using analytical C18 RP-HPLC. Edman degradation analysis yielded signals for alkylated Cys residues (Pth-CM-Cys) in cycles 5 and 16, indicative of 5-to-16 and 10-to-23 assignments of the disulfide bridge framework in RhTx. (e) An ensemble of 10 RhTx structures, with the disulfide bridges shown in blue.

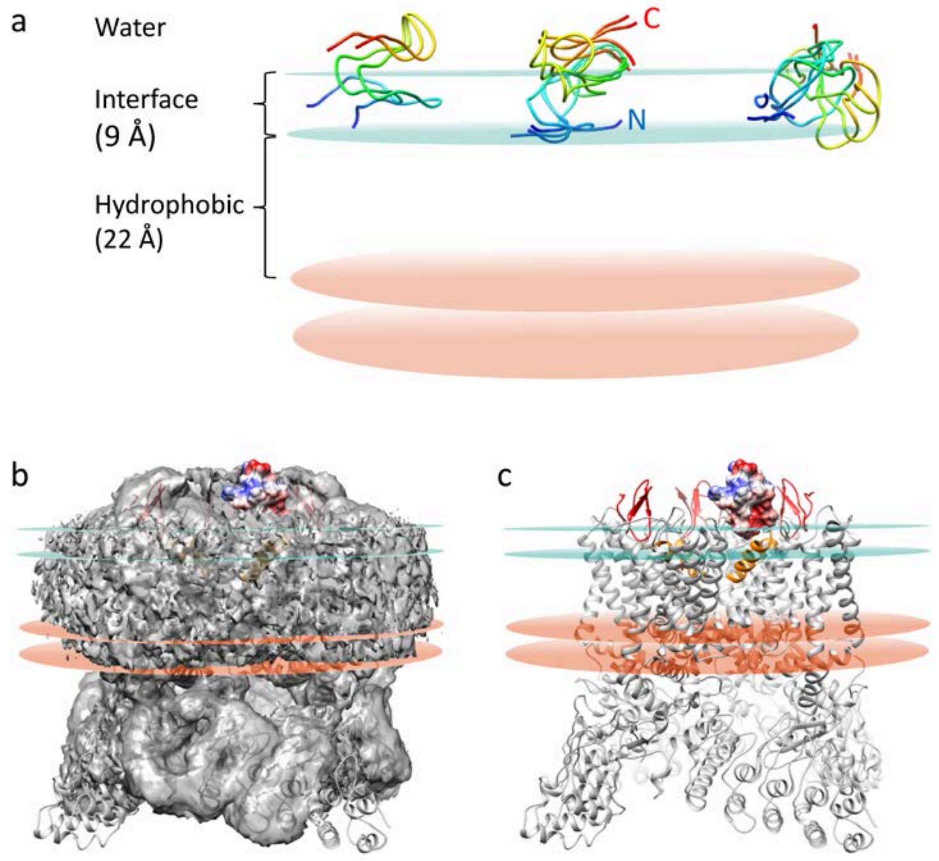

Supplementary Figure 5. Interactions between RhTx and lipid membrane probed by computation. (a) Implicit membrane environment is setup as the hydrophobic, interface and water layers in Rosetta. RhTx participates mainly within the interface (defined by two cyan planes) between hydrophobic core of lipid membrane and aqueous environment. Three clusters of the 10 NMR structure models of RhTx are observed after scoring by RosettaMembrane energy function. RhTx are rainbow colored by sequence. The uncharged N terminus (blue) of RhTx points towards the hydrophobic layer of membrane, while the charged C terminus (red) stays in water. (b) The same implicit membrane environment as shown in (a) is mapped onto the cryo-EM density map of TRPV1 (EMD ID: 5776, contoured at level 3). The observed electron density of amphipols, which indicate the transmembrane region of TRPV1, distributes mostly within this implicit membrane environment. The surface of RhTx colored by electrostatic potential is shown. (c) Structural model of TRPV1 in complex with RhTx (colored electrostatic potential surface) and DkTx (red ribbons).

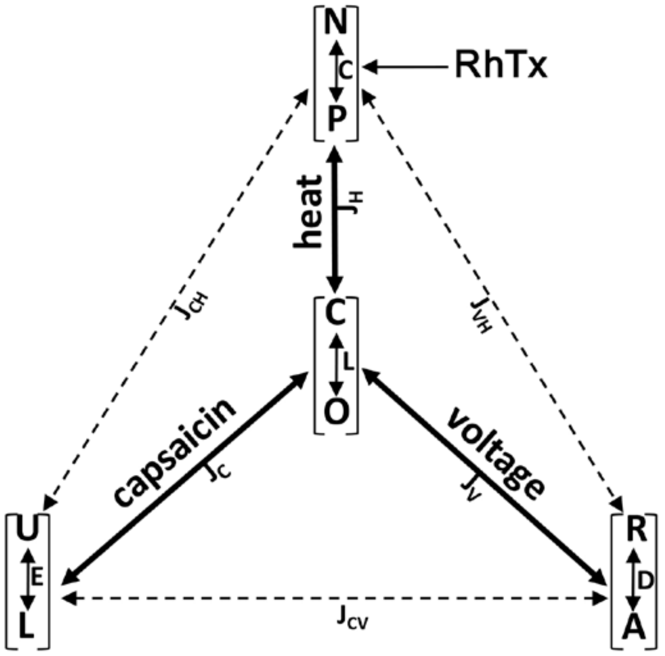

Supplementary Figure 6. An allosteric model for predicting agonist response to cooling. In this generic model, channel activators capsaicin, voltage, and heat are assumed to promote channel pore opening C←→O by allosteric coupling through distinct pathways, as described in Experimental Procedures. Detail descriptions of model parameters and calculations are given in a recent report[1].

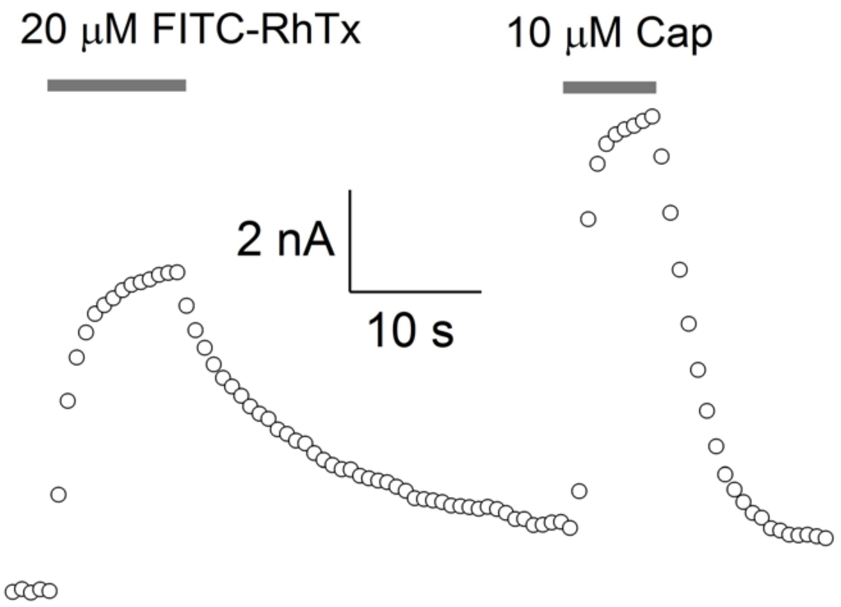

Supplementary Figure 7. FITC-RhTx activates TRPV1. Representative whole-cell recording of TRPV1 channels at +80 mV activated by RhTx labeled with FITC at its N terminus. 10 μM Capsaicin was applied as a positive control.

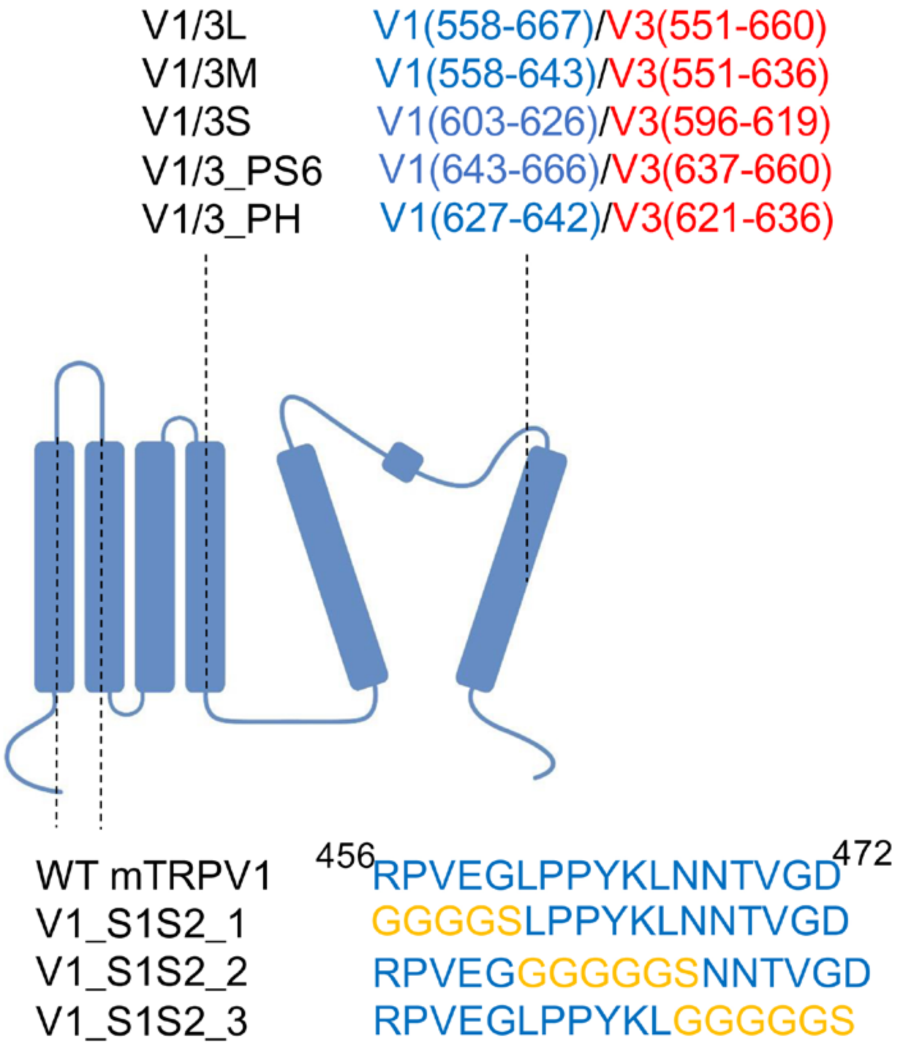

Supplementary Figure 8. Designs of the TRPV1/TRPV3 chimeric channels and the S1-S2 linker sequence replacement mutants.

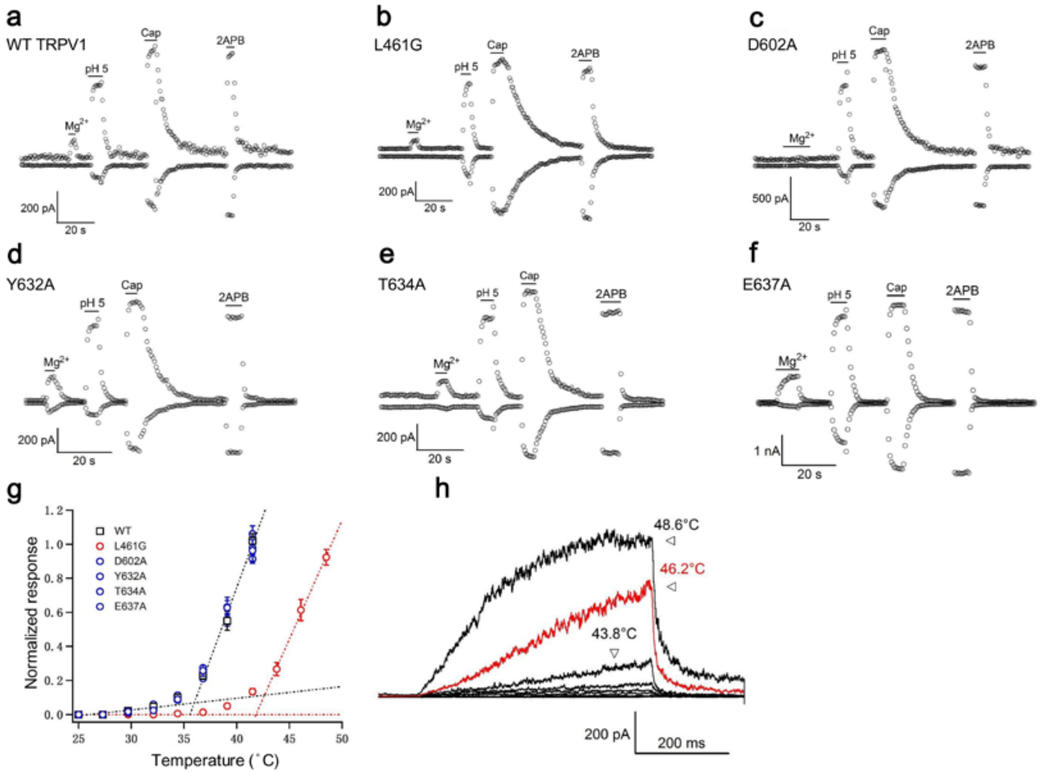

Supplementary Figure 9. TRPV1 point mutants retain near-normal gating functions. (a-f) Outside-out patch recordings demonstrating activation of WT mTRPV1 or point mutants by 130 mM Mg^{2+}, proton (pH 5.0), 10 μM capsaicin and 3 mM 2APB. (g) Heat-induced activation of WT mTRPV1 and point mutants. While all the channels were heat-activated, L461G exhibited a significant shift in the activation threshold temperature to a higher temperature comparing to WT or the other mutants, suggesting that the mutation affected the normal heat activation process. The observation is fully consistent with reports that sequence perturbations to the S1-S2 linker could disrupt Mg^{2+}- and heat-dependent activation[1] and H^{+}-dependent activation[2] of TRPV1, as well as voltage-dependent activation of Kv channels[3]. (h) Outside-out patch recordings of L461G activation induced by laser heating.

Supplementary Table 1. Example animal peptide toxins and their lengths.

Name	Length (amino acids)
Spider double-knot toxin, DkTx	75
Snake α-bungarotoxin, α-BTx	74
Scorpion charybdotoxin, CTx	37
Spider hanatoxin, HaTx	35
Cone snail conotoxins	12 to 30
Centipede red-head toxin, RhTx	27

Supplementary Table 2. NMR and refinement statistics for RhTx.

	RhTx
NMR distance & dihedral constraints	
Distance constraints	
Total NOE	216
Intra-residue	105
Inter-residue	111
Sequential (|i–j| = 1)	69
Medium-range (|i–j|<4)	13
Long-range (|i–j|>5)	29
Intermolecular	0
Hydrogen bonds	0
Total dihedral angle restraints	15
phi	15
Psi	0
Structure Statistics	
Violations (mean and s.d.)	
Distance constraints (Å)	0.041 ± 0.005

Dihedral angle constraints (°)	0.948 ± 0.162
Max. dihedral angle violation (°)	1.38
Max. distance constraint violation (Å)	0.314
Deviations from idealized geometry	
Bond (Å)	0.004 ± 0.0002
Angles (°)	0.568 ± 0.048
Improper (°)	1.475 ± 0.201
Average pairwise r.m.s.d. ** (Å, 2rd structure)	
Heavy	1.29 ± 0.21
Backbone	0.75 ± 0.22

** Pairwise r.m.s.d. was calculated among 20 refined structures.

Supplementary References

1 Cao, X., Ma, L., Yang, F., Wang, K. & Zheng, J. Divalent cations potentiate TRPV1 channel by lowering the heat activation threshold. *The Journal of general physiology* **143**, 75-90, doi:10.1085/jgp.201311025 (2014).

2 Boukalova, S., Teisinger, J. & Vlachova, V. Protons stabilize the closed conformation of gain-of-function mutants of the TRPV1 channel. *Biochimica et biophysica acta* **1833**, 520-528, doi:10.1016/j.bbamcr.2012.11.017 (2013).

3 Lee, S. Y., Banerjee, A. & MacKinnon, R. Two separate interfaces between the voltage sensor and pore are required for the function of voltage-dependent K(+) channels. *PLoS biology* **7**, e47, doi:10.1371/journal.pbio.1000047 (2009).
